# LSP Methylation in Skeletal Muscle and Cross-Tissue Candidate NUMT Signals Reveal Complementary Features of Mitonuclear Aging in Mice

**DOI:** 10.3390/biology15211971

**Published:** 2026-11-02

**Authors:** Xijia Wang, Wenpeng Li, Xingbo Zhao

**Affiliations:** State Key Laboratory of Animal Biotech Breeding, College of Animal Science and Technology, China Agricultural University, Beijing 100193, China

**Keywords:** aging, mitochondrial-nuclear crosstalk, LSP methylation, NUMT, *Ptprc*

## Abstract

Aging disrupts communication between mitochondria and the nucleus, but compact molecular readouts of this interface remain limited. Here, we examined mitochondrial DNA light-strand promoter (LSP) methylation signals in skeletal muscle across natural-aging and mitochondrial-background models, and independently profiled candidate nuclear mitochondrial DNA insertion (NUMT) junction signals in brain and skeletal muscle across age groups. Natural aging was associated with reduced motor performance, altered skeletal-muscle mitochondrial status, and lower LSP methylation signals. Reciprocal F1 crosses and conplastic mice showed coordinated associations among mitochondrial background, LSP methylation signals, bioenergetic parameters, nuclear mitochondrial-gene expression, and physical performance. In parallel, short-read whole-genome sequencing revealed a non-monotonic candidate NUMT signal pattern, with the highest mean signal at 12 months. A *Ptprc*-associated candidate mitochondrial-nuclear junction was supported by split-read inference and junction-specific droplet digital PCR in a candidate-positive sample. Together, these findings identify LSP methylation signals and candidate NUMT junction signals as complementary but independently measured molecular features of mitonuclear aging.

## Simple Summary

Aging is accompanied by changes in mitochondrial function and genome stability, but simple molecular features that reflect communication between mitochondria and the nucleus remain limited. In this study, we examined two complementary features in mice: methylation signals in a regulatory region of mitochondrial DNA and candidate signals of mitochondrial DNA fragments associated with the nuclear genome. We found that aging and different mitochondrial genetic backgrounds were associated with changes in mitochondrial DNA methylation signals, mitochondrial status, and physical performance. In a separate genome analysis, candidate mitochondrial–nuclear junction signals were most abundant in middle-aged mice rather than increasing steadily with age. One candidate junction located within the *Ptprc* gene region was further supported by a targeted DNA assay in a candidate-positive sample. These findings suggest that mitochondrial DNA regulatory changes and candidate mitochondrial–nuclear junction signals may provide complementary information about changes occurring during aging, although further studies are needed to determine their biological relationship and functional significance.

## 1. Introduction

Aging is characterized by declining physiological resilience, impaired tissue homeostasis, and increased risk of chronic disease. Chronological age, however, incompletely captures functional vitality, motivating the development of molecular markers that report biological aging states [1,2,3,4]. Among the hallmarks of aging, mitochondrial dysfunction and genome instability are tightly connected. Somatic mutation and DNA damage studies have reinforced genome instability as a core aging process [5,6,7]. Mitochondrial defects alter ATP production, redox balance, innate immune signaling, and stress responses, whereas nuclear genome instability can compromise mitochondrial maintenance and cellular identity [8,9,10].

Mitochondria are integrated genetically and functionally with the nucleus. Most mitochondrial proteins are nuclear-encoded, whereas mitochondrial DNA (mtDNA) encodes core oxidative phosphorylation components and provides stress-responsive signals. Fitness therefore depends on compatibility between mitochondrial haplotypes and nuclear genetic backgrounds, a principle supported by studies linking mitonuclear matching to metabolism, disease expressivity, and healthy aging [11,12,13]. Practical readouts of this compatibility remain limited.

One candidate readout is the methylation signal within the mtDNA light-strand promoter (LSP) region. Because mitochondrial transcription is strand-asymmetric and the LSP defines initiation of light-strand transcription, locus-resolved analysis of this regulatory region may provide a focused readout of mitochondrial regulatory state. However, mtDNA methylation remains technically and biologically debated, with studies reporting either low or absent CpG methylation or context-dependent methylation signals [14,15,16,17,18,19]. The LSP is nonetheless a compact regulatory region involved in mtDNA transcription and replication [20,21]. We therefore use locus-resolved LSP methylation signals as a candidate molecular readout without interpreting them as a measure of global mtDNA methylation.

Mitonuclear remodeling may also appear as structural genome instability. Nuclear mitochondrial DNA segments (NUMTs) arise when mtDNA fragments are incorporated into nuclear chromosomes, and both inherited and somatic NUMTs have been documented across eukaryotic and human genomes [22,23,24,25,26]. Mitochondrial-to-nuclear DNA transfer can occur during double-strand break repair and end-joining [27,28,29,30,31,32], and mitochondrial stress can release mtDNA into the cytosol to promote inflammatory signaling [33,34,35,36,37,38,39].

Aging follows nonlinear molecular trajectories, including stage-like proteomic and multi-omic transitions [40,41]. Such nonlinear changes suggest that aging-associated molecular features may exhibit transient or stage-specific patterns rather than monotonic accumulation. We therefore asked whether LSP methylation signals and candidate NUMT junction signals provide complementary information about mitonuclear aging. Because these features were measured in partially independent experimental cohorts, we analyzed them as parallel molecular correlates rather than as directly linked components of a single aging trajectory.

## 2. Materials and Methods

### 2.1. Animals and Experimental Design

Male C57BL/6J mice were used for young (3 months) and aged (18 months) comparisons. ICR and C57BL/6J mice were crossed reciprocally to generate F1 offspring with comparable autosomal composition and distinct maternally inherited mtDNA haplotypes. Reciprocal F1 offspring were designated as IC (ICR male × C57BL/6J female) and CI (C57BL/6J male × ICR female), respectively. Because mitochondrial DNA is maternally inherited, these reciprocal crosses generate offspring with comparable autosomal compositions but distinct mitochondrial genomes, enabling assessment of mitochondrial-background-associated differences while not excluding maternal, parent-of-origin, or sex-chromosome effects. For conplastic analysis, female NZB/OlaHsd mice were crossed with male C57BL/6J mice, and female offspring carrying NZB mtDNA were backcrossed to C57BL/6J males for 13 generations to generate BL6^NZB^ mice. BL6^C57^ controls carried C57BL/6J nuclear and mitochondrial genomes. Candidate NUMT analysis used brain and skeletal muscle WGS data from male C57BL/6J mice at 3, 12, and 18 months; young and mid-life groups contained three biological replicates each, and the aged group contained six biological replicates. Mice were maintained under specific-pathogen-free conditions at 25 °C with a 12 h light/dark cycle and ad libitum access to chow and water. Procedures were reviewed and approved by the Laboratory Animal Welfare and Animal Experimental Ethics Committee of China Agricultural University.

For the natural-aging comparison, behavioral and biochemical phenotyping was performed using 10 male mice per group, LSP methylation analysis using five mice per group, and whole-genome sequencing using three mice per group.

For the reciprocal-F1 comparison, behavioral and biochemical phenotyping was performed using 10 male mice per group, LSP methylation analysis using five mice per group, and RNA sequencing using three mice per group. Reciprocal F1 mice were phenotyped and sampled at 6 months of age.

For the conplastic comparison, behavioral and biochemical phenotyping was performed using 10 male mice per group, and LSP methylation analysis using five mice per group. BL6^NZB^ and BL6^C57^ mice were phenotyped and sampled at 6 months of age.

Random allocation was not applicable because experimental groups were defined by age, reciprocal-cross direction, or mitochondrial genetic background. Investigators were not blinded to group allocation during the experiments or data analysis.

### 2.2. Behavioral Assessment of Motor Function

Motor endurance was measured using a rotarod test at 40 rpm. Each mouse completed four trials with 10 min inter-trial intervals, and the longest latency to fall was analyzed. Forelimb muscle strength was measured with a grip-strength meter. Mice gripped a horizontal grid connected to a force gauge and were gently pulled backward by the tail until release. Peak force was recorded across repeated trials, and the maximum value was used for analysis.

### 2.3. Mitochondrial Biochemical Assays and mtDNA Copy Number

ATP and ROS levels in skeletal muscle were quantified using commercial kits according to the manufacturers’ protocols. ATP content was measured spectrophotometrically, and ROS was quantified using DCFH-DA fluorescence. Total genomic DNA was extracted from muscle tissue for mtDNA copy-number analysis by SYBR Green quantitative PCR on a LightCycler 480 system. Relative mtDNA copy number was calculated as 2^−ΔCt^, where ΔCt = Ct (mtDNA target) − Ct (nuclear reference target), using measurements from the same DNA sample. This calculation provides a nuclear-DNA-normalized index of mtDNA abundance for comparisons among samples, consistent with established qPCR quantification principles and mtDNA/nDNA analysis methods. The calculation assumes comparable amplification efficiencies close to 100% for the mitochondrial and nuclear targets. Values were not further normalized to a control-group calibrator and therefore represent relative mtDNA abundance rather than fold changes against a control group or calibrated absolute copy numbers per cell. Primers are listed in Table S1.

### 2.4. Whole-Genome Resequencing and Variant Calling

Genomic DNA from muscle samples of young and aged mice was sequenced at approximately 30× depth. Libraries with 200–300 bp inserts were sequenced on the DNBSEQ platform. Raw reads were filtered with SOAPnuke (v2.1.9), aligned to the mouse reference genome with BWA-MEM (v0.7.19), and sorted and indexed with SAMtools (v1.24). Alignment quality was assessed with Qualimap2 (v2.3). Variants were called using a GATK GVCF-based joint-genotyping workflow. SNPs and insertion/deletions were filtered and annotated with SnpEff; copy-number variation and genome-wide burden summaries were generated as in the source analysis.

### 2.5. mtDNA LSP Methylation Analysis

For all natural-aging, reciprocal-F1, and conplastic comparisons, LSP methylation was measured using DNA extracted from skeletal muscle. Bisulfite conversion was performed using the EZ-96 DNA Methylation-Gold Kit. Primers targeting the mouse mtDNA LSP region were designed against the reference mitochondrial sequence. Sequence comparison confirmed that the primer-binding regions and all analyzed cytosine positions within the amplified LSP region were conserved among the C57BL/6J, ICR, and NZB mitochondrial haplotypes. Bisulfite-converted amplicons were sequenced on an Illumina MiSeq platform. Adapter and primer sequences were removed with Cutadapt, paired-end reads were merged with PEAR, quality was assessed with FastQC, and cleaned reads were aligned to a bisulfite-converted mouse mitochondrial reference using Bismark. Methylation at each cytosine site was calculated as the percentage of methylated reads among total reads at that position. Bisulfite conversion efficiency exceeded 99.1%. Given the ongoing debate regarding mtDNA methylation, we interpret these measurements as locus-resolved LSP methylation signals rather than as an estimate of global mtDNA methylation. Although the high conversion efficiency provides quality control for bisulfite conversion, it does not resolve the broader technical debate surrounding mtDNA methylation detection. Orthogonal validation will therefore be valuable in future studies. Primer and cycling information are provided in Table S2.

Because methylation measurements were performed from bulk skeletal muscle DNA, the resulting values represent tissue-level LSP methylation signals and may integrate contributions from different cellular and mitochondrial populations.

### 2.6. RNA Sequencing and qPCR Validation

For the reciprocal F1 comparison, total RNA was extracted from skeletal muscle of three mice per group. cDNA libraries were sequenced on the BGISEQ-500 platform. High-quality reads were aligned to GRCm39 using Bowtie2, and expression was estimated with RSEM and normalized as FPKM. Differentially expressed genes were defined using fold change ≥ 2 and false discovery rate ≤ 0.001, followed by KEGG enrichment. Selected mitochondrial-related nuclear genes in skeletal muscle of BL6^NZB^ and BL6^C57^ mice with three biological replicates per group, including *Ppargc1b*, *Cox6a1*, *Cox8a*, *Slc25a5*, *Ucp1*, and *Aco1*, were validated by qPCR and normalized to beta-actin using the 2^−ΔΔCt^ method.

### 2.7. Validation of Conplastic Nuclear Background and mtDNA Origin

WGS reads from BL6^NZB^ mice were aligned to GRCm39. Diagnostic SNPs fixed between C57BL/6NJ and NZB/B1NJ reference strains were obtained from Mouse Genome Project data, and approximately 200,000 autosomal SNPs were used to estimate nuclear-genome similarity. Genotypes at diagnostic sites were called from BAM pileups using a custom Python (v3.14.7) workflow. NZB mtDNA origin was verified by PCR amplification and Sanger sequencing of a mitochondrial fragment containing an informative SNP distinguishing NZB from C57BL/6J mtDNA.

### 2.8. Candidate NUMT Detection and Annotation

Brain and skeletal-muscle WGS reads were aligned to the GRCm39/mm39 reference genome using minimap2 with the short-read parameters -ax sr -Y --secondary=yes. Alignments were sorted and indexed using SAMtools. To identify reference NUMTs and reduce false-positive candidate calls, the mouse mitochondrial genome was aligned against the nuclear genome using BLASTN (version 2.12.0+) with dust filtering disabled, a word size of 11, and an E-value threshold of 1 × 10^−10^. High-scoring segment pairs with an alignment length of at least 50 bp and sequence identity of at least 80% were retained. Adjacent hits separated by no more than 250 bp in either nuclear or mitochondrial coordinates were merged, and merged reference NUMT loci shorter than 80 bp were excluded. The resulting reference NUMT intervals were used as exclusion regions during sample-level candidate detection.

Candidate mitochondrial-to-nuclear junction loci were identified from each BAM file using a custom chimeric-read-based workflow. Discordant read pairs and split or soft-clipped reads supporting nuclear-mitochondrial junctions were extracted and clustered according to their nuclear breakpoint coordinates. Supporting alignments were required to have a mapping quality of at least 20, and soft-clipped segments were required to be at least 20 bp long. Supporting reads located within 500 bp were grouped into the same candidate locus. A candidate locus was retained when it was supported by at least three reads in total, including at least one split read. Supporting evidence was summarized by read name to avoid counting multiple alignments from the same read pair more than once. Candidate loci located within 500 bp of an annotated reference NUMT were excluded. Sample-level candidate loci were subsequently merged across the cohort using a 500 bp merging window, and loci detected in at least one sample were retained in the cohort-level candidate set.

Cohort-level candidate loci were quantified separately in each sample. Breakpoint-supporting reads were counted within a 500 bp window surrounding each candidate locus, and local sequencing depth was estimated from the 250 bp flanking intervals. Normalized support was calculated as the number of breakpoint-supporting reads divided by the mean sequencing depth of the flanking intervals. A locus was considered detected in a sample when it was supported by at least three reads in total, including at least one split read. To identify age-associated NUMT loci, normalized support values were compared among Young, Mid-life, and Aged groups within each tissue using the non-parametric Kruskal–Wallis test. Loci with no variation across samples were excluded from statistical testing, and *p* values were adjusted for multiple testing using the Benjamini–Hochberg method. Candidate NUMT loci with an adjusted *p* value < 0.05 were considered significantly altered across age groups.

Genomic annotation was performed with the GRCm39/mm39 GTF file. Promoters were defined as 2000 bp upstream to 500 bp downstream of transcription start sites, and loci were classified as promoter, intronic, or intergenic. Gene Ontology Biological Process and KEGG enrichment analyses were performed using gseapy/Enrichr and treated as exploratory after inspection of overlapping genes.

### 2.9. Ptprc Junction Inference and ddPCR Validation

Supporting reads from a mid-life brain candidate-positive sample were reanalyzed to infer the *Ptprc*-associated candidate NUMT junction (Table S3). Nuclear and mitochondrial alignments were clustered to define the breakpoint and mtDNA source interval. Junction-specific primers and a hydrolysis probe were designed to detect the predicted *Ptprc*-mtDNA junction rather than mtDNA or wild-type *Ptprc* alone (Table S4). A 500-fold diluted candidate-positive DNA sample was tested in a 20-uL ddPCR reaction using Bio-Rad ddPCR Supermix for Probes and the QX200 system. FAM-positive droplets were classified using a consistent amplitude threshold. Candidate-negative young and aged brain DNA controls were tested under the same assay.

### 2.10. Quantification and Statistical Analysis

Data are presented as mean ± SEM, mean ± SD, or mean with 95% confidence intervals as indicated. Two-group comparisons used unpaired two-tailed Student’s *t* tests when assumptions of approximate normality and comparable variance were met; otherwise, Mann–Whitney U tests were used. Multiple-group comparisons used one-way ANOVA with Tukey post hoc tests for parametric data or Kruskal–Wallis tests with Dunn-type post hoc comparisons for non-parametric data. Site-resolved methylation and related endpoint families were adjusted with Benjamini–Hochberg false-discovery-rate correction where applicable. Age-associated candidate NUMT support differences were evaluated with Kruskal–Wallis tests and Benjamini–Hochberg correction. Exact sample sizes are indicated by data points in figures where applicable.

## 3. Results

### 3.1. Natural Aging Is Associated with Altered Mitochondrial Status and Reduced LSP Methylation Signals

We first tested whether physiological aging is accompanied by coordinated changes in motor performance, mitochondrial status, and LSP methylation signals. Relative to young C57BL/6J mice, aged mice showed shorter rotarod latency and lower forelimb grip strength, indicating reduced endurance and muscle function (Figure 1A,B). These organismal phenotypes were accompanied by reduced ATP content and mtDNA copy number and elevated reactive oxygen species (ROS) in skeletal muscle (Figure 1C–E).

Under the present reference-based variant-calling workflow, aged mice showed a higher number of SNP, indel, and CNV calls relative to the reference genome than young mice (Figure 1F,G and Figure S1). These results represent differences in reference-differing variant-call burden and should not be interpreted as direct measurements of age-acquired somatic mutations. Site-resolved bisulfite sequencing revealed reduced LSP methylation signals in aged mice, with differential methylation patterns detected at individual cytosine positions within the LSP region (Figure 1H,I). Thus, aged mice exhibited reduced physical performance, altered mitochondrial status, and lower LSP methylation signals.

### 3.2. Reciprocal F1 Crosses Show Mitochondrial-Background-Associated Differences in LSP Methylation

To determine whether LSP methylation reports mitonuclear context rather than chronological age alone, we analyzed reciprocal F1 hybrids generated from ICR and C57BL/6J mice. Reciprocal F1 offspring have comparable autosomal compositions but inherit distinct maternal mitochondrial backgrounds. IC denotes offspring from ICR males × C57BL/6J females and therefore carries C57BL/6J mtDNA, whereas CI denotes offspring from C57BL/6J males × ICR females and carries ICR mtDNA. Differences between the reciprocal groups are therefore consistent with mitochondrial-background effects, although maternal, parent-of-origin, and sex-chromosome effects cannot be excluded (Figure 2A).

The reciprocal groups diverged in mitochondrial-associated biochemical traits and physiological phenotypes. Offspring carrying the C57BL/6J mitochondrial haplotype had higher skeletal-muscle ATP content and mtDNA copy number, lower ROS, longer rotarod latency, and greater grip strength than the reciprocal group carrying the ICR mitochondrial haplotype (Figure 2B–F). These differences were accompanied by higher overall LSP methylation signals and increased signals at multiple individual cytosine positions within the LSP region (Figure 2G–I). Selected nuclear-encoded mitochondrial-related and metabolic regulatory genes showed coordinated expression differences between the reciprocal F1 groups (Figure 2J). These genes were grouped into functional categories including oxidative phosphorylation/electron transport (*Cox6a1* and *Cox8a*), mitochondrial biogenesis and metabolic regulation (*Ppargc1b* and *Pparg*), mitochondrial transport and membrane-associated functions (*Slc25a5* and *Tspo*), metabolic and redox-associated processes (*Aco1*, *Nsdhl*, and *Cndp1*), stress/adaptive regulation (*Jund* and *Ucp1*), and cell-cycle regulation (*Ccnd2* and *Ccnd3*). These data demonstrate coordinated differences in LSP methylation signals, transcriptional profiles, mitochondrial-associated traits, and physiological performance between the reciprocal F1 groups.

### 3.3. Conplastic Mice Associate Mitochondrial Background with LSP Methylation and Physiological Performance

Reciprocal crosses reduce, but do not remove, genetic complexity. We therefore used conplastic mice to examine the association of a defined mtDNA background with phenotypic and molecular traits on a predominantly C57BL/6J-like nuclear background. NZB mtDNA was introgressed onto the C57BL/6J nuclear background through 13 generations of backcrossing, generating BL6^NZB^ mice; BL6^C57^ mice served as controls (Figure 3A). Nuclear-genome similarity analysis confirmed a highly C57BL/6J-like nuclear background in BL6^NZB^ mice, and Sanger sequencing verified the NZB mtDNA origin (Figure 3B,C).

BL6^NZB^ mice showed higher skeletal-muscle ATP content and mtDNA copy number, lower ROS, and higher rotarod and grip-strength performance relative to BL6^C57^ controls (Figure 3D–H). BL6^NZB^ mice also showed higher overall LSP methylation signals and increased signals at multiple cytosine positions across the LSP region (Figure 3I,J). qPCR confirmed higher expression of several mitochondrial-related nuclear genes (Figure 3K). Together with the reciprocal F1 experiment, these results support an association between mitochondrial-nuclear background, LSP methylation, and physiological phenotypes.

### 3.4. Candidate NUMT Signals Show a Mid-Life-High Pattern and Include a Ptprc-Associated Candidate Junction

We next examined whether an independent structural-genomic dataset revealed additional candidate signatures associated with mitonuclear remodeling during aging. Because LSP methylation signals and candidate NUMT signals were not measured as paired endpoints across the same complete experimental design, this analysis was treated as a complementary structural-genomic layer rather than a direct mechanistic extension of the LSP findings.

Approximately 30× short-read whole-genome sequencing data from brain and skeletal muscle were analyzed in young, mid-life, and aged mice using a chimeric-read workflow that excluded annotated reference NUMTs. We first examined all detected candidate NUMT loci, regardless of their statistical significance across age groups. At this global detection level, the mean number of candidate NUMT loci was highest at mid-life in both tissues and lower in young and aged animals. These detected loci were distributed across multiple chromosomes, with broader chromosomal representation in mid-life samples, and most were located in intergenic or intronic regions (Figure 4A,B and Figure S2A). Thus, the overall candidate-locus landscape showed a non-monotonic detection pattern, indicating a mid-life-high detection pattern in the present cohort rather than a monotonic age-associated pattern. This pattern describes the temporal distribution of candidate NUMT signal detection in this cohort and does not by itself establish the biological mechanisms responsible for the mid-life enrichment.

We then focused specifically on candidate NUMT loci that showed significant age-associated quantitative changes. For each tissue, normalized breakpoint support was compared among young, mid-life, and aged groups, and loci passing multiple-testing correction were defined as significant age-associated candidate NUMT loci. This significance-filtered subset generally followed a young-low, mid-life-high, aged-low trajectory (Figure 4C–J). Among these loci, the *Ptprc*-associated candidate junction represented one of the most interpretable shared intragenic candidate signals detected across brain and skeletal muscle. Exploratory annotation of mid-life candidate loci produced immune-related terms, but inspection of overlapping genes showed that this signal was driven primarily by the *Ptprc*-associated intronic candidate locus rather than by broad multi-gene immune pathway enrichment (Figure S3). However, these annotations do not establish that the candidate junctions occurred within immune cells or that immune pathways directly drive NUMT formation. Split-read reanalysis of a mid-life brain sample placed the nuclear breakpoint between NC_000067.7:138084627 and NC_000067.7:138084628 within a *Ptprc* intron and was consistent with a putative insertion of an approximately 190 bp mtDNA fragment from NC_005089.1:3109-3298 in reverse-complement orientation (Figure 4L, Table S3). Junction-specific droplet digital PCR detected the predicted *Ptprc*-mtDNA junction in the candidate-positive sample but not in candidate-negative young or aged brain controls (Figure 4M). This result supports detectability of the predicted junction sequence but does not quantify its allele fraction or establish the complete insertion architecture.

## 4. Discussion

Mitochondrial dysfunction and genome instability are central and interconnected hallmarks of aging [4,7,8,10,12]. Recent evidence highlights progressive alterations in mitochondrial function as important contributors to aging-associated physiological decline and metabolic imbalance [42,43]. In the present study, LSP methylation signals were associated with mitochondrial background, mitochondrial-associated traits, and physical performance, whereas candidate NUMT junction signals showed a non-monotonic, mid-life-high pattern in brain and skeletal muscle. Together, these observations identify two complementary but independently measured molecular features of mitonuclear aging. Because the two features were assessed in partially independent cohorts, the present study does not establish a direct or mechanistic relationship between them.

The LSP findings are consistent with previous evidence that mitochondrial haplotypes and nuclear backgrounds can interact to influence metabolism and physiological phenotypes [11,12,13]. Site-resolved analysis identified differential cytosine positions within the LSP region, but the present study does not establish that individual sites directly regulate mitochondrial transcription. Moreover, because only the LSP region was examined, it remains unclear whether the observed pattern is specific to LSP or reflects broader mtDNA epigenetic variation. These considerations are particularly important given the continuing debate over the existence, abundance, and interpretation of mtDNA methylation [14,15,16,17]. By combining natural-aging, reciprocal-F1, and conplastic-mouse comparisons, the present study shows that mitochondrial background is associated with differences in LSP methylation signals, bioenergetic parameters, and physiological performance.

However, because these measurements were obtained from bulk skeletal muscle tissue, the contribution of cell composition and mitochondrial population heterogeneity cannot be fully excluded. Although ATP content, ROS levels, and mtDNA copy number provide complementary indicators of mitochondrial status, they do not capture all aspects of mitochondrial bioenergetic function, such as membrane potential and respiratory capacity. Future studies incorporating membrane potential and oxygen consumption measurements will further define the functional consequences of mitochondrial background variation.

The candidate NUMT data provide a distinct structural-genomic observation. In this cross-sectional cohort, candidate mitochondrial-nuclear junction signals were highest at mid-life, consistent with reports that molecular features of aging can follow nonlinear trajectories [40,41,44]. Age-resolved profiling of mouse organs has identified linear and nonlinear expression trajectories involving immune and inflammatory processes, with immune-cell activation detectable in white adipose tissue by middle age [45]. Separately, a reporter-mouse study found lower non-homologous end-joining efficiency in cells from old mice than in those from young mice [46]. These findings provide context for age-dependent immune remodeling and changes in DNA repair, but do not establish a mid-life peak in repair activity or demonstrate that either process explains the candidate NUMT pattern observed in our cohort. The biological basis of this pattern remains unresolved, and the present data do not determine whether it reflects changes in junction formation, cellular composition, clonal representation, or other age-dependent factors. Because detection relies primarily on short-read WGS, these loci should be interpreted as candidate mitochondrial-nuclear junction signals rather than confirmed structural insertions. Long-read sequencing or targeted structural validation will be required to determine their complete architecture and prevalence [47,48].

The *Ptprc*-associated candidate mitochondrial-nuclear junction is biologically intriguing but requires cautious interpretation. *Ptprc* encodes CD45, a receptor-type protein tyrosine phosphatase involved in immune-cell identity and signaling [49,50]. Because *Ptprc* is an immune-associated locus, variation in immune-cell representation within bulk tissue provides a plausible alternative explanation for the detected signal. Bulk-tissue WGS cannot determine whether the junction originates from resident parenchymal cells, infiltrating immune cells, or a rare cell population. Therefore, the current data establish the presence of a *Ptprc*-associated candidate junction in the analyzed sample but do not establish its cellular origin or mechanism of generation.

## 5. Limitations of the Study

Several limitations define the scope of this study. First, LSP methylation and candidate NUMT signals were measured in partially independent cohorts, precluding within-animal correlation and causal inference between the two molecular features. Second, LSP methylation was assessed by bisulfite sequencing within a single mtDNA regulatory region using bulk skeletal muscle. Therefore, the present study cannot establish LSP specificity, exclude contributions from cellular or mitochondrial heterogeneity, or determine whether individual methylation sites are functionally causal. Orthogonal methylation assays and cell-resolved analyses will be required to address these questions. Third, candidate NUMT loci were inferred primarily from short-read WGS. Although reference NUMTs and low-confidence chimeric alignments were filtered and one *Ptprc*-associated junction was supported by ddPCR, the complete insertion architecture, allele fraction, and cellular origin remain unresolved; the ddPCR assay establishes junction detectability rather than quantitative tissue-wide burden. Fourth, ATP, ROS, and mtDNA copy number describe complementary aspects of mitochondrial status but do not directly measure membrane potential or respiratory capacity. Finally, the mid-life-high candidate NUMT pattern is cross-sectional, and its biological basis remains unresolved.

## 6. Conclusions

This study identifies LSP methylation signals and candidate mitochondrial–nuclear junction signals as two complementary but independently measured molecular features associated with mitonuclear aging. LSP methylation signals varied with natural aging and mitochondrial genetic background and were accompanied by differences in mitochondrial-associated traits and physical performance. In a separate structural-genomic analysis, candidate mitochondrial–nuclear junction signals showed a non-monotonic, mid-life-high pattern in brain and skeletal muscle, and a *Ptprc*-associated candidate junction was further supported by split-read inference and junction-specific ddPCR in a candidate-positive sample. These findings do not establish a direct mechanistic relationship between LSP methylation and candidate NUMT formation, nor do they establish the complete structural architecture of the candidate junctions. Future studies combining paired measurements, orthogonal methylation assays, and long-read or targeted structural validation will be needed to determine the biological relationship and functional significance of these molecular features during aging.

## Figures and Tables

**Figure 1:**
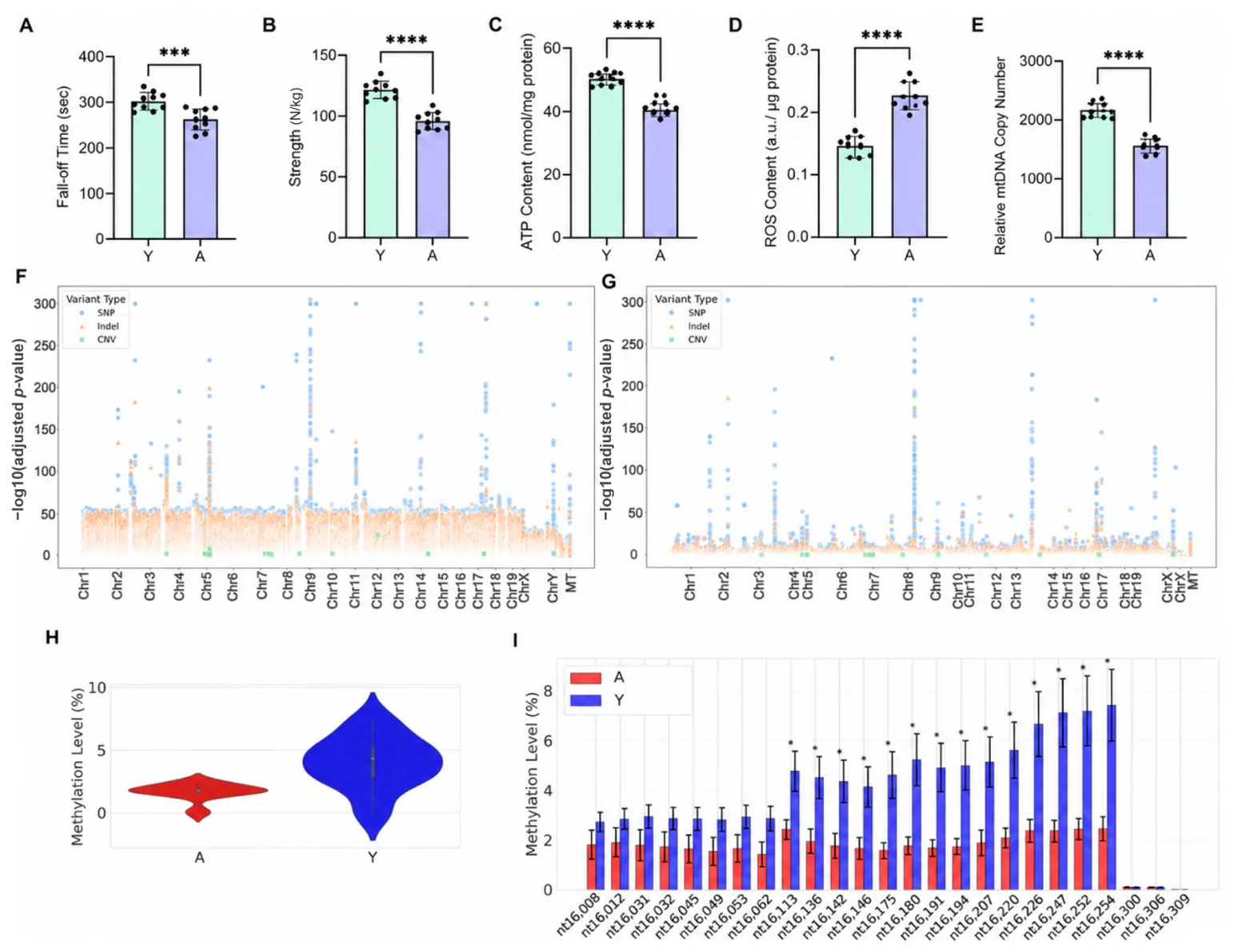
Aging is associated with physiological decline, altered mitochondrial status, differences in reference-based variant-call burden, and reduced mtDNA LSP methylation. (**A**,**B**) Rotarod endurance and forelimb grip strength in young (Y) and aged (A) mice. (**C**–**E**) Skeletal-muscle ATP content, ROS level, and relative mtDNA copy number. In (**E**), relative mtDNA copy number is expressed as 2^−ΔCt^ following normalization to the nuclear DNA reference, without additional normalization to the young-group mean. (**F**,**G**) Genome-wide variant burden identified by whole-genome resequencing in aged and young mice relative to the reference genome. (**H**,**I**) Overall and site-resolved methylation across the mitochondrial light-strand promoter (LSP) region. Data are mean ± SEM; statistical tests are described in Materials and methods. * *p* < 0.05, *** *p* < 0.001, **** *p* < 0.0001.

**Figure 2:**
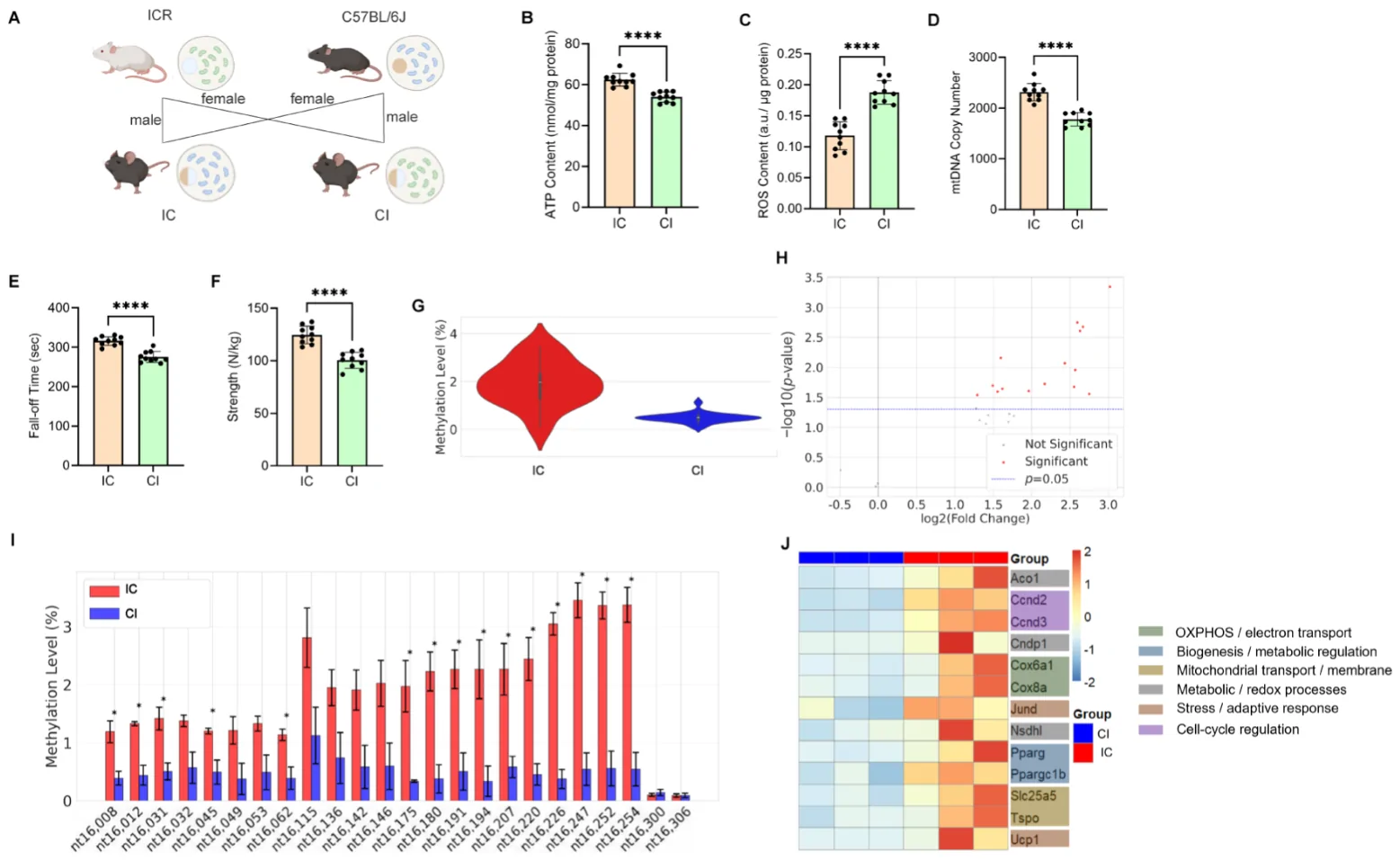
Reciprocal F1 crosses reveal mitochondrial haplotype-associated physiological, mitochondrial, epigenetic, and transcriptional outcomes. (**A**) Reciprocal-crossing design between ICR and C57BL/6J mice. IC denotes ICR male × C57BL/6J female offspring, whereas CI denotes C57BL/6J male × ICR female offspring. (**B**–**D**) ATP content, ROS level, and relative mtDNA copy number in skeletal muscle. (**E**,**F**) Rotarod endurance and forelimb grip strength. (**G**–**I**) Overall and site-resolved LSP methylation. (**J**) Expression profile of nuclear-encoded mitochondrial-related and metabolic regulatory genes in skeletal muscle. Genes are annotated by functional category, including oxidative phosphorylation/electron transport, mitochondrial biogenesis/metabolic regulation, mitochondrial transport/membrane-associated function, metabolic/redox processes, stress/adaptive response, and cell-cycle regulation. Data are mean ± SEM; statistical tests are described in Materials and methods. * *p* < 0.05, **** *p* < 0.0001.

**Figure 3:**
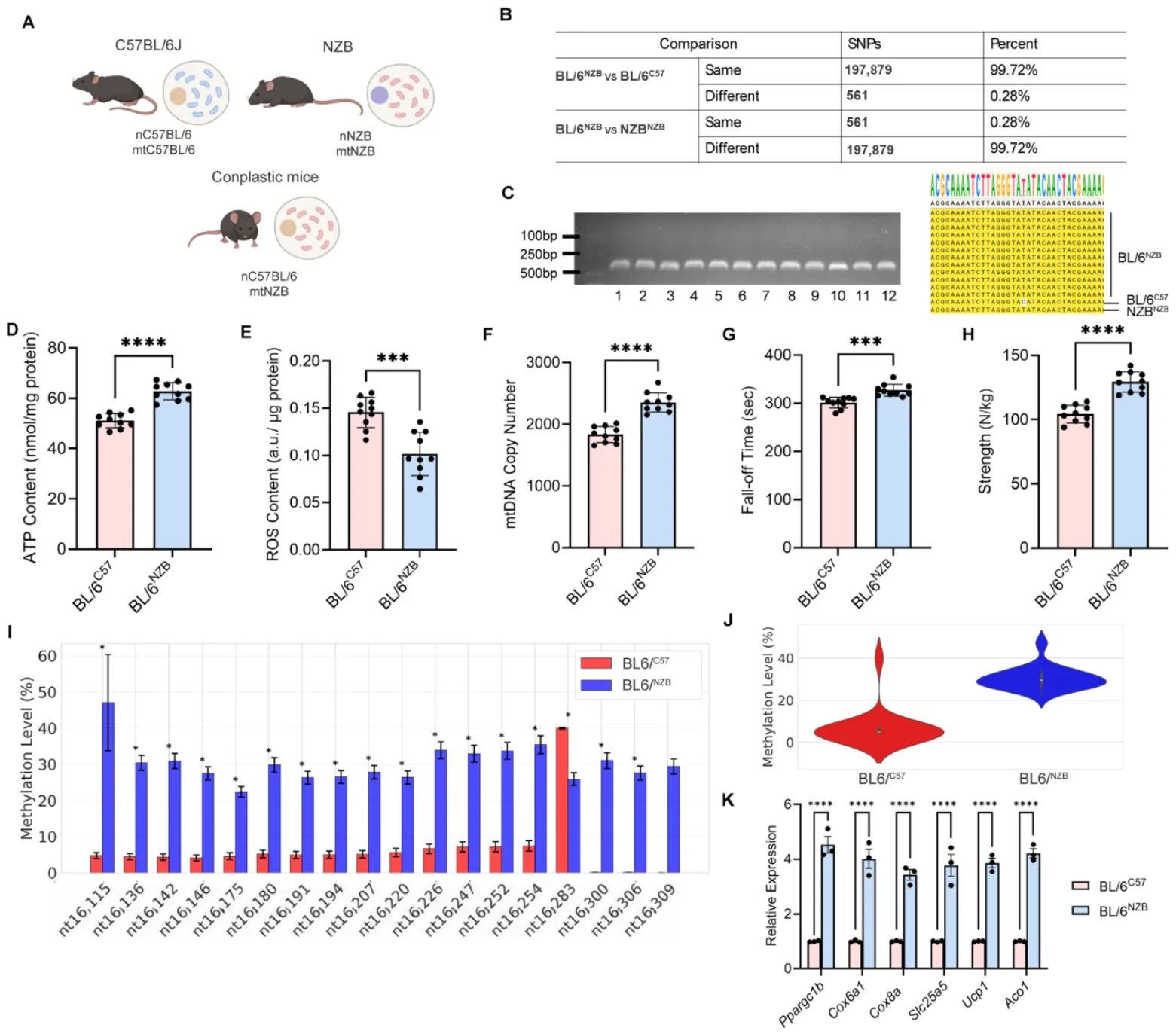
Conplastic mitochondrial background is associated with LSP methylation and physiological performance. (**A**) Generation of BL6^NZB^ mice by repeated backcrossing of NZB mtDNA onto a C57BL/6J nuclear background. (**B**,**C**) Nuclear background similarity and mtDNA-origin validation. (**D**–**F**) ATP content, ROS level, and relative mtDNA copy number. (**G**,**H**) Rotarod endurance and grip strength. (**I**,**J**) Site-resolved and overall LSP methylation. (**K**) qPCR validation of mitochondrial-related gene expression. Data are mean ± SEM; statistical tests are described in Materials and methods. * *p* < 0.05, *** *p* < 0.001, **** *p* < 0.0001.

**Figure 4:**
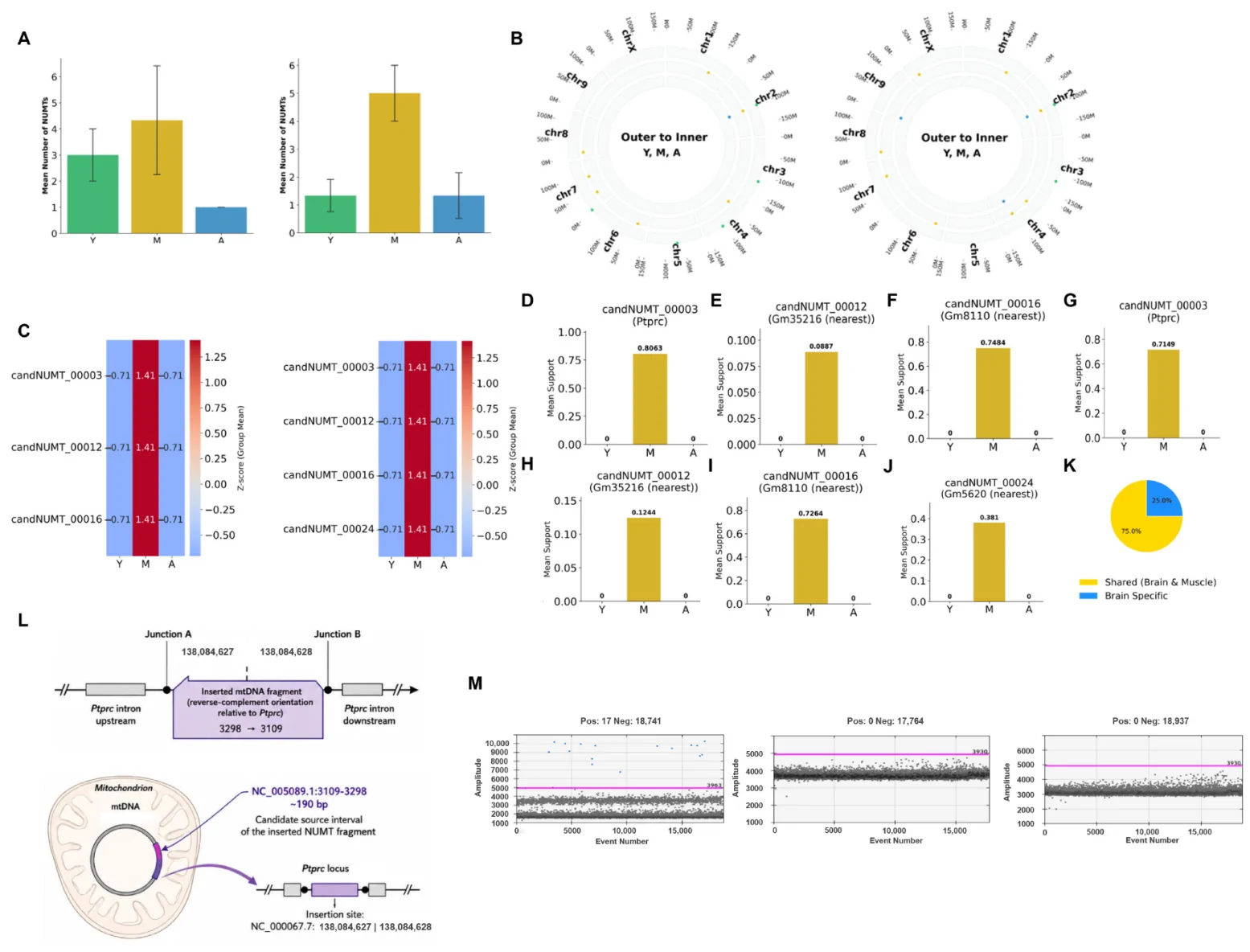
Candidate NUMT signals show a mid-life-high pattern and identify a *Ptprc*-associated candidate mitochondrial-nuclear junction signal. (**A**) Overall candidate NUMT counts in brain and muscle. (**B**) Genome-wide candidate-locus distributions in brain and muscle. (**C**) Heatmaps of significant age-associated candidate NUMT loci in brain and muscle. (**D**–**F**) Mean normalized support for significant age-associated candidate NUMT loci and associated genes in brain. (**G**–**J**) Mean normalized support for significant age-associated candidate NUMT loci and associated genes in muscle. (**K**) Tissue-sharing pattern. (**L**) Predicted *Ptprc*-mtDNA junction model. (**M**) Junction-specific ddPCR. WGS-based calls represent candidate NUMT junction signals derived from short-read sequencing and should be considered screening-level events unless independently validated.

## Data Availability

The data that support the findings of this study are available on request from the corresponding author.

## Supplementary Materials

The following supporting information can be downloaded at: https://www.mdpi.com/article/10.3390/biology15211971/s1, Figure S1: Genome-wide variant burden in aged mice relative to the reference genome. (A)The numbers of SNPs, insertion/deletions (InDels), and copy-number variants (CNVs) identified in young (Y) mice relative to the reference genome. (B)The numbers of SNPs, InDels, and CNVs identified in aged (A) mice relative to the reference genome. Figure S2: Genomic feature distribution of candidate NUMT loci. (A) Genomic feature distribution of overall candidate NUMT loci in brain and muscle. (B) Genomic feature distribution of significant age-associated candidate NUMT loci in brain and muscle. Figure S3: *Ptprc*-centered immune-related annotation of mid-life candidate NUMT-associated loci. (A and B) Gene Ontology Biological Process terms associated with NUMT-linked genes in brain and muscle. (C and D) KEGG pathway terms in brain and muscle. Enrichment results are exploratory and should be interpreted as *Ptprc*-centered functional annotation rather than broad multi-gene pathway activation. Table S1: Primer sequences used for mtDNA copy-number analysis; Table S2: LSP bisulfite PCR primers and cycling conditions; Table S3: Split-read-supported inference of the *Ptprc*-associated NUMT junction architecture. Table S4: Sequences of primers and probes in the junction ddPCR.
