# Supplementary material for: LSP Methylation in Skeletal Muscle and Cross-Tissue Candidate NUMT Signals Reveal Complementary Features of Mitonuclear Aging in Mice
Source: Biology (Basel). 2026 Nov 2;15(21):1971. doi: 10.3390/biology15211971 (PMC13762117; doi:10.3390/biology15211971)
Supplement: Supplementary file 1 [file biology-15-01971-s001.zip › biology-4543429-supplementary.pdf]

Supplemental information

LSP Methylation in Skeletal Muscle and Cross-Tissue Candidate NUMT Signals Reveal Complementary Features of Mitonuclear Aging in Mice

Xijia Wang, Wenpeng Li, and Xingbo Zhao

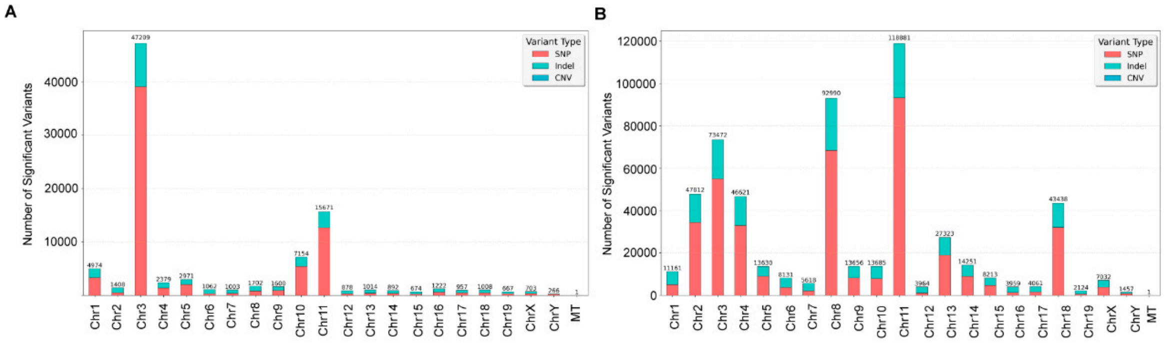

Figure S1. Genome-wide variant burden in aged mice relative to the reference genome. (A)The numbers of SNPs, insertion/deletions (InDels), and copy-number variants (CNVs) identified in young (Y) mice relative to the reference genome. (B)The numbers of SNPs, InDels, and CNVs identified in aged (A) mice relative to the reference genome.

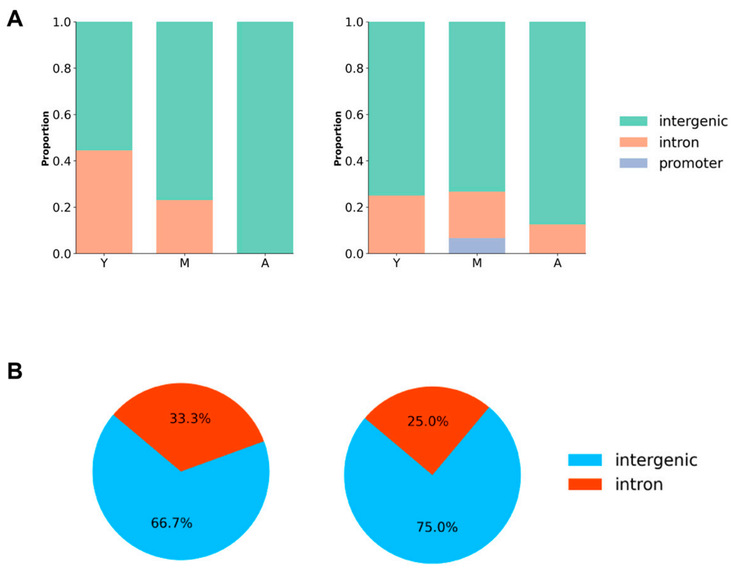

Figure S2. Genomic feature distribution of candidate NUMT loci. (A) Genomic feature distribution of overall candidate NUMT loci in brain and muscle. (B) Genomic feature distribution of significant age-associated candidate NUMT loci in brain and muscle.

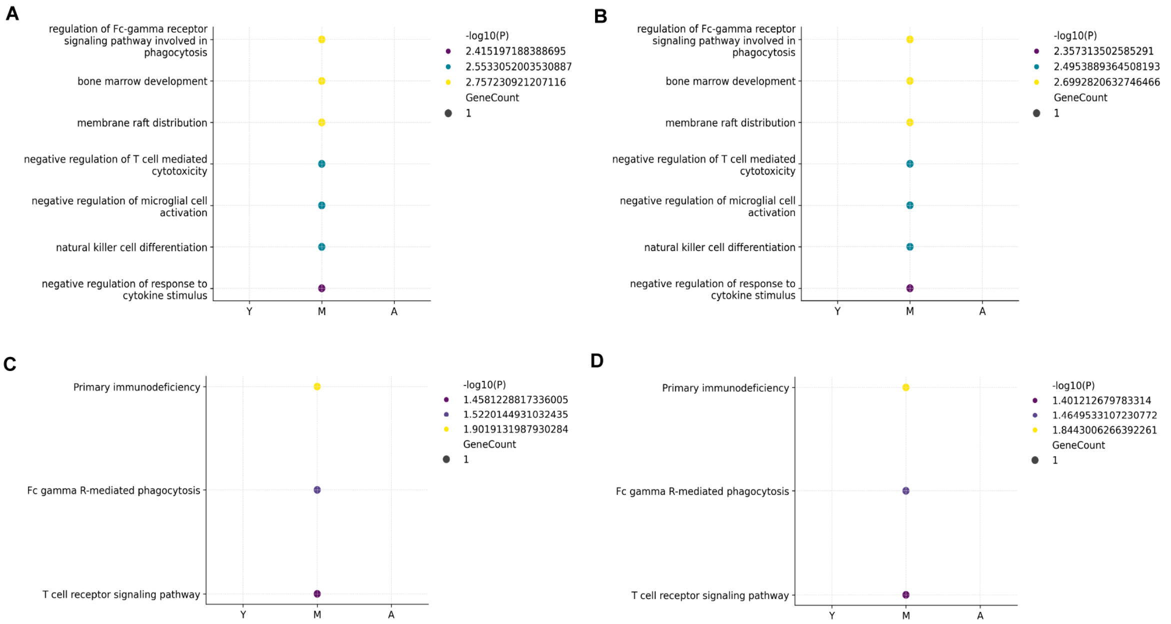

Figure S3. Ptprc-centered immune-related annotation of mid-life candidate NUMT-associated loci. (A and B) Gene Ontology Biological Process terms associated with NUMT-linked genes in brain and muscle. (C and D) KEGG pathway terms in brain and muscle. Enrichment results are exploratory and should be interpreted as *Ptprc*-centered functional annotation rather than broad multi-gene pathway activation.

Table S1. Primer sequences used for mtDNA copy-number analysis

Primer name	Primer sequence (5' to 3')	Product size (bp)
MND1	F: TTGGTCCATACGGCATTT<br>R: GAGTGATAGGGTAGGTGC	119
MACTB	F: GCCTTCCTTCTTGGGTAT<br>R: GTCTTTACGGATGTCAACG	88

Table S2. LSP bisulfite PCR primers and cycling conditions

Item	Sequence or condition	Product size/time
Forward primer	TATTAATTTGTTAATGTTTATTGCGTAA	442 bp
Reverse primer	CGAATCTAATCAACCCATAACCA	442 bp
Initial denaturation	95°C	3 min
Denaturation	94°C	30 s
Annealing	55°C	30 s
Extension	72°C	45 s
Cycles	Steps 2-4	35 cycles
Final extension	72°C	5 min

Table S3. Split-read-supported inference of the Ptprc-associated NUMT junction architecture

Parameter	Result
Representative sample	Mbrain2
Selected cluster	cluster_01451
Ptprc cluster region	NC_000067.7:138084346–138084926
Supporting read names	51
Dominant mtDNA contig	NC_005089.1
Dominant mtDNA strand support	+
Reads supporting best mtDNA interval	37
Alignment records supporting best mtDNA interval	61
mt_start_median	3139
mt_end_median	3269
mtDNA source interval	NC_005089.1:3109–3298
Estimated mtDNA fragment length	~190 bp
Inferred Ptprc insertion site	NC_000067.7:138084627|138084628
Inferred orientation relative to Ptprc	Reverse-complement
Junction A	Ptprc 138084627 ↔ mtDNA 3298
Junction B	mtDNA 3109 ↔ Ptprc 138084628
Orthogonal validation	Junction-specific ddPCR positive droplets detected

Table S4. Sequences of primers and probes in the junction ddPCR

name	Sequence 5'→3'	Length	Tm	GC%
*Ptprc*_NUMT_A_F	GGAAACTATATCATTTCTAAGTGGG	25	59.6°C	36.0
*Ptprc*_NUMT_A_R	CTCTCTACAAACACTTATTACAACC	25	60.0°C	36.0
*Ptprc*_NUMT_A_Probe	TTAAAGATGGCTATGGGTCAGGCTGG	26	68.9°C	50.0
